# Insights into the Evolutionary Features of Human Neurodegenerative Diseases

**DOI:** 10.1371/journal.pone.0048336

**Published:** 2012-10-30

**Authors:** Arup Panda, Tina Begum, Tapash Chandra Ghosh

**Affiliations:** Bioinformatics Centre, Bose Institute, Kolkata, India; Kyushu Institute of Technology, Japan

## Abstract

Comparative analyses between human disease and non-disease genes are of great interest in understanding human disease gene evolution. However, the progression of neurodegenerative diseases (NDD) involving amyloid formation in specific brain regions is still unknown. Therefore, in this study, we mainly focused our analysis on the evolutionary features of human NDD genes with respect to non-disease genes. Here, we observed that human NDD genes are evolutionarily conserved relative to non-disease genes. To elucidate the conserved nature of NDD genes, we incorporated the evolutionary attributes like gene expression level, number of regulatory miRNAs, protein connectivity, intrinsic disorder content and relative aggregation propensity in our analysis. Our studies demonstrate that NDD genes have higher gene expression levels in favor of their lower evolutionary rates. Additionally, we observed that NDD genes have higher number of different regulatory miRNAs target sites and also have higher interaction partners than the non-disease genes. Moreover, miRNA targeted genes are known to have higher disorder content. In contrast, our analysis exclusively established that NDD genes have lower disorder content. In favor of our analysis, we found that NDD gene encoded proteins are enriched with multi interface hubs (party hubs) with lower disorder contents. Since, proteins with higher disorder content need to adapt special structure to reduce their aggregation propensity, NDD proteins found to have elevated relative aggregation propensity (RAP) in support of their lower disorder content. Finally, our categorical regression analysis confirmed the underlined relative dominance of protein connectivity, 3′UTR length, RAP, nature of hubs (singlish/multi interface) and disorder content for such evolutionary rates variation between human NDD genes and non-disease genes.

## Introduction

The pathogenesis of neuronal vulnerability in neurodegenerative diseases (NDD), involving amyloid formation in specific brain regions, is still not clear. Therefore, tracing evolutionary etiology of human misfolding and aggregation related disease genes can shed light into the molecular mechanism of neurodegenerative disease pathogenesis by identifying the factors that harbor disease causing mutations in normal genes. Thus, we used *Homo sapiens* as model organism to assess the molecular divergence of neurodegenerative diseases by computing the ratio of number of non-synonymous substitution per site (*d*N) to the number of synonymous substitution per site (*d*S) against non-disease genes as the control parameter [1].

Decades-long continuous efforts have facilitated to characterize protein evolutionary rates with the signatures of gene expression level [2], [3], protein length [4], [5], aggregation propensity [6], [7], number of interacting partners [8], miRNA targets [9], gene dispensability [10], [11] and protein disorder content [12], [13]. Due to lack of proper 3D structure, protein intrinsically disordered region provide global flexibility that promotes binding to their partners in protein-protein interactome [14], [15]. Moreover, highly connected miRNA targeted genes are highly disordered in nature [13]. On the other hand, exposed hydrogen bonds in highly disordered proteins are known to elevate the risk of protein aggregation, which may impose selective constraints on protein structures [13], [16]. Hence, to resolve the ambiguity of relations affecting protein evolutionary rates, we specifically analyzed human aggregation prone neurodegenerative disease genes compare to non-disease genes. We considered protein intrinsic disorder content, miRNA targeting and protein connectivity as the functions of evolutionary rates.

Finally, our comprehensive analysis revealed the conserve nature of human NDD genes relative to non-disease genes. We exploited several evolutionary parameters to explain the slower evolutionary rates of NDD genes with respect to non-disease genes. Moreover, we here obtained lower disorder content in NDD genes, conflicting the previously established analyses of Roychoudhury et al. [17] and Uversky [18]. Relative importance of the determinants in modulating evolutionary rates of proteins was further confirmed from categorical regression analysis which emphasized that protein connectivity, 3′UTR length, relative aggregation propensity (RAP), nature of hubs (singlish/multi-interface) and disorder content are largely responsible for such evolutionary behaviour of human NDD genes. Furthermore, we also confirmed that the nature of hub is also an important evolutionary rate regulator.

## Materials and Methods

### Dataset Preparation for Evolutionary Rate Estimation

We listed 460 non-redundant NDD genes from Biobase Knowledge Library (BKL) (http://www.biobase-international.com/) out of 848 readily available NDD annotations by matching their functional description with any one of the neurodegenerative diseases common to literatures (such as Alzheimer disease, Parkinson disease, Huntington disease, Adrenoleukodystrophy, Creutzfeldt-Jakob disease, Friedreich ataxia, Leigh syndrome, Neuronal Ceroid lipofuscinosis, Myoclonic epilepsy, Pick disease, Spinocerebellar ataxia, Supranuclear palsy, Charcot-Marie-tooth disease, Wolfram syndrome, Alexander disease, Amyotrophic lateral sclerosis, Canavan disease, Familial dysautonomia, Leukoencephalopathy, Metachromatic leukodystrophy, Multiple sclerosis, Myotonic dystrophy, Prion diseases, Rett syndrome, Schizophrenia, Spastic paraplegia, Spinal muscular atrophy, Multiple system atrophy and Tay-sachs disease) [19]–[34]. However, some of the afore-mentioned diseases may belong to neuropathy or lysosomal storage disease groups [35], [36] and were excluded from our gene set. To extract actual disease genes, we also removed potential risk associated disease susceptible genes as per Online Mendelian Inheritance in Man (OMIM) [37], Human Gene Mutation Database (HGMD) [38] and Genetic Association Database (GAD) [39] from our dataset. In our comparative study, genes not showing any disease annotation in BKL or OMIM or HGMD or GAD and did not follow ubiquitous expression pattern [40], were regarded as non-disease genes. Following 1∶1 orthology relationship [41], we extracted the corresponding mouse orthologues of the human genes from Ensembl v.60 using biomart [42] and also obtained their pairwise non-synonymous (*d*N) and synonymous (*d*S) substitution rates to compute gene specific evolutionary rate (*d*N/*d*S). Genes having *d*S >3 were discarded from our analysis to get rid of problems due to mutational saturation [43]. Human protein coding sequences were also acquired from Ensembl database. For genes with more than one isoform, the longest isoform was considered. Finally, we yielded a list of 375 NDD and 7578 non-disease genes with available evolutionary rate for further analysis (Table S1).

### Determining Gene Expression Level and Expression Width

Following the method of Wu et al. [44], we estimated gene expression level using HG-U133A affymetrix probe set in addition to the GNF1B, GCRMA dataset obtained from Gene Expression Atlas (http://biogps.gnf.org/downloads/). An average intensity value in 84 tissues was considered as the expression level for each gene. In case of genes with different probe sets, we averaged the mean expression values of all the probe sets of a gene to yield final gene expression level [7]. Gene expression width is determined as per Park et al. [45], where, we took a cutoff signal intensity value as 200 to consider a gene is expressed in that particular tissue. We, thereby, obtained expression data for 356 NDD genes and 3930 non-disease genes.

### Protein-Protein Interaction Data

Human protein-protein interaction data was collected from biological interaction repository BioGRID database v.3.1.77 [46] which houses over 10271 unique human proteins annotated with 39931 non-redundant interactions. BioGRID acts as an extensive interaction pool compare to other human interaction databases like HPRD, MIPS, FlyBase etc [47]–[49]. Therefore, for systematic analysis of interaction network, we chose BioGRID database to compute protein connectivity by counting the number of interaction partners (excluding self interaction) that a protein connects with.

### Identification of Nature of Hub Proteins (Singlish/Multi Interface Hub)

Hub proteins can be characterized by the proteins with ≥5 interactors [50]. As per Kim et al. [51], we have assigned the hub proteins as singlish/multi interface hubs by identifying their interacting domains using Pfam database [52]. To assign a domain, the following criteria were used: (a) e-value of alignment should be <10^−4^, (b) protein sequence should overlap >80% of the domain length and (c) length of the domain should be greater than 12 residues [51]. Following Kim et al. [50], [51] we annotated hub proteins with one or two interacting interface as singlish interface hub and those having more than two interacting interfaces as multi interface hubs.

### microRNA Targeting and 3′ UTR Length Calculation

Human miRNA target predictions were obtained from microRNA.org database (August’2010 releases) [53]. We only considered miRNAs, whose target sites remain conserved across the mammalian phylogeny, to acquire a reliable outcome [13]. Using the prediction, we next computed the number of regulatory miRNAs per gene in our dataset. Ensembl v.60 [42] was used to calculate the length of 3′UTR region for each gene.

### Estimation of Protein Disorder Content

In our dataset, we predicted intrinsic disorder of a protein using versatile graphic web server FoldIndex (http://bioportal.weizmann.ac.il/fldbin/findex) [54] using its default parameters. To reduce false positive rate only the sequences with 30 or more disordered residues at a stretch were considered [12], [55]. The fraction of disorder content was estimated by dividing the number of disordered residues of a protein to the length of that protein.

### Computing Protein Relative Aggregation Propensity (RAP)

Aggregation propensity of both NDD and non-disease proteins was retrieved using TANGO algorithm [56]. Based on the physicochemical properties, TANGO predicts the β-aggregation score of a protein. To calculate RAP of a protein, we took the ratio of its TANGO aggregation score to the maximal TANGO aggregation score of the whole dataset [7], [57].

### Statistical Analyses

The entire statistical analyses were performed using SPSS v.13. Mann-Whitney U test was used to compare the average values of different variables between two classes of genes. For correlation analysis, we performed the Spearman’s Rank correlation coefficient ρ, where the significant correlations were denoted by *P*<0.05.

## Results

### Gene Expression Level Constraining the Evolutionary Rates of NDD Genes

Neurodegenerative diseases are known to be arisen through complex interaction between genetics of a given individual and multiple environmental factors [58]. Therefore, studying evolutionary aspect of progressive degenerative diseases of the central nervous systems has enormous impact on evolutionary genetics, which led us to estimate the evolutionary rates (*d*N/*d*S) of 375 neurodegenerative disease and 7578 non-disease genes in our comparative analysis. We observed that NDD genes are under purifying selection pressure as compare to non-disease genes (*d*N/*d*S of NDD genes = 0.126, non-disease genes = 0.158 and *P = *1.90×10^−6^ for NDD vs. non-disease genes). Therefore, to illuminate the conserved nature of NDD genes, we computed gene expression levels of both NDD and non-disease genes as expression levels are known to be the major evolutionary rates indicator [59]. Reasonably, we noticed that mean expression levels of NDD genes (94.338) are ∼2.54 fold higher than non-disease genes (Expression level = 37.057, *P = *3.31×10^−7^ for NDD vs. non-disease genes).

Moreover, we obtained a strong negative correlation between gene expression levels and protein evolutionary rates (Spearman’s ρ = −0.108, *P* = 1.00×10^−6^). Hence, it can be concluded that gene expression levels to be one of the potential evolutionary features responsible for such rate variations.

### Examining Protein Connectivity and miRNA Targeting as Influential Factors of Protein Evolutionary Rates

Proteins with higher interacting partners evolve slower as mutations in protein interaction sites may disrupt the network connectivity affecting the functionality of the proteins [8], [60]–[63]. Hence, considerable lower evolutionary rates of NDD genes in contrast to non-disease genes directed us to scrutinize whether protein connectivity has any influence on their evolutionary rates differences. We found that highly expressed NDD genes encoding proteins have ∼2 fold higher network connectivity in comparison with non-disease genes encoding proteins (average connectivity of NDD proteins = 10.59, non-disease proteins = 5.71, *P = *1.95×10^−14^ for NDD vs. non-disease proteins). Additionally, in agreement to Fraser et al. [8], [61] a significant negative correlation is detected between protein connectivity and evolutionary rates (Spearman’s ρ = −0.162, *P* = 1.00×10^−6^). Thus, we infer that protein connectivity may have an impact on evolutionary rate differences between NDD and non-disease genes.

It is now obvious that highly connected proteins are targeted by greater number of miRNAs because genes targeted by various types of miRNAs are subject to enormous functional constraints and thus, evolve slowly [13]. Therefore, retrieving miRNA targets against each gene revealed that NDD genes are highly targeted by various types of miRNAs compare to the non-disease genes (mean miRNA targets of NDD gene = 44.88, non-disease gene = 39.44, *P = *1.11×10^−3^ for NDD vs. non-disease genes). Moreover, miRNAs can recognize target sites at the 3′UTR regions of the genes and hence genes with longer 3′UTR evolve at slower rates compare to genes with shorter 3′UTR [9]. Estimation of the 3′UTR length of NDD and non-disease genes (Mean 3′UTR length of NDD gene = 1749 bp, non-disease gene = 1536 bp, *P = *2.72×10^−2^ for NDD vs. non-disease genes) also supports the earlier results [9]. Correlation analysis revealed that evolutionary rate is negatively correlated with the number of distinct miRNA types (Spearman’s ρ = −0.087, *P* = 1.00×10^−6^) and also with 3′UTR length (Spearman’s ρ = −0.192, *P* = 1.00×10^−6^). Thus, our results emphasize that number of miRNA types and 3′UTR length altogether modulate the rate difference between NDD and non-disease genes.

### Protein Intrinsic Disorder Content and Nature of Hub Proteins as the Functions of Protein Evolutionary Rates

**Figure 1:**
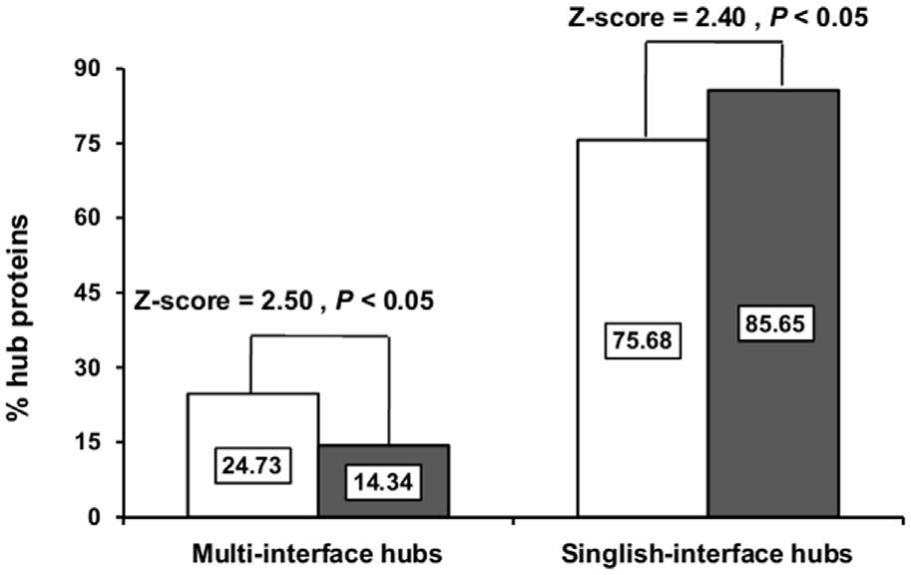
Multi-interface proteins are prevalent in NDD genes compare to non-disease genes. The bar diagram depicts the percentage of hub proteins in NDD and non-disease genes within singlish and multi-interface hubs respectively. In each group, the dark bar represents non-disease genes whereas other bar belongs to NDD category.

Genes encoding proteins with higher intrinsically disorder regions (IDRs) are targeted by higher number of miRNAs rather than genes encoding proteins with lower IDRs [13]. Therefore, it is expected that highly connected NDD genes should have greater disorder content than non-disease genes, as observed earlier [17], [18]. Interestingly, our observation contrasts our expectation i.e. NDD genes have significantly lower disorder content (21.98%) than non-disease genes (25.98%) (*P = *5.23×10^−3^ for NDD vs. non-disease proteins). In favor of our observation, we also found a significant positive association (ρ = 0.080, *P* = 1×10^−6^) between IDR content and *d*N/*d*S. In addition, it is well known that highly disordered proteins serve as flexible linkers in the protein-protein interaction networks to promote promiscuous binding to their interacting partners [64], [65]. Since, we observed a greater connectivity of NDD genes compare to non-disease genes; it is expected that NDD genes should have higher disorder content than the non-disease genes as observed previously [17]. Moreover, highly connected “hub” proteins (with ≥5 interactors) in the protein-protein interaction network play a vital role in controlling biological processes of cell [66]. Surprisingly, we observed that NDD genes have greater proportion of hub proteins than non-disease genes (Table 1). Previously, it has been reported that multi interface hubs (party hubs) interact simultaneously with their partners and exhibit relatively conserved evolutionary rates with lower disorder content than singlish interface hubs (date hubs) that facilitate transient binding with their different partners at different times/locations [12], [50], [51]. Moreover, due to lack of compact 3-D structures in native state, intrinsically disorder proteins are under less structural constraint and have elevated evolutionary rates [13]. Accordingly, we found that NDD genes are enriched with multi interface hubs (party hubs) (Figure 1) in favor of their lower disorder content and also supports for their lower evolutionary rate compare to non-disease genes.

**Table 1:** Proportions of hub proteins in NDD and non-disease gene encoded proteins with different cutoff values for interaction partners.

Hub contents in differentconditions	NDD proteins Vs. Non-diseaseproteins (Respectively)	Z score	Significance Level
With partner ≥5	50.000% vs. 33.880%	5.497	99.9%
With partner ≥10	31.292% vs. 15.599%	6.803	99.9%
With partner ≥20	14.966% vs. 5.760%	6.028	99.9%

*Note*. 100% confidence level refers to significance level: *P*<0.01.

### Relative Aggregation Propensity Negatively Steers Protein Evolutionary Rates

Earlier it has been reported that the frequency of aggregation nucleating segments is significantly lower in intrinsically disordered proteins compare to properly folded proteins. These results have been explained due to lack of structural constraints in intrinsically disordered proteins which finally safeguards proteins against aggregation [6], [67]–[69]. This led us to measure the RAP of each individual protein in our dataset using TANGO algorithm [7], [56]–[57]. We found that NDD genes encoded proteins are highly aggregation prone with respect to non-disease gene encoded proteins (average RAP of NDD proteins = 0.097, non-disease proteins = 0.083, *P = *9.21×10^−6^ for NDD vs. non-disease proteins). Moreover, we found an overall negative correlation between RAP and percentage of intrinsically disordered residues (ρ = −0.467, P = 1×10^−6^) and between RAP and evolutionary rates (ρ = −0.072, P = 1×10^−6^). Thus, we propose that RAP also regulate the evolutionary rates of NDD and non-disease genes.

### Independent Forces of Protein Evolutionary Rates Using Categorical Regression Model

We have identified that gene expression level, number of miRNAs targeting the gene, 3′UTR length, percentage of intrinsically disordered residues, number of interacting partners, natures of hub (i.e. singlish interface hub/multi interface hub) and RAP are the attributes regulating the evolutionary rates of the NDD genes with respect to non-disease genes. In order to excavate the independent influence of the above mentioned six predictor variables on protein evolutionary rates, we performed categorical regression analysis to best predict the value of the dependent variable as categorical regression can optimally scale the categorical data to its numerical equivalents [70]. According to our ANOVA model (*F = *13.648, *P*<0.05), protein connectivity, 3′UTR length, RAP, nature of hubs (singlish/multi interface) and disorder content were found to be the independent evolutionary rate modulators (Table 2).

**Table 2:** Categorical regression to illustrate the independent influential evolutionary features.

Parameter	Standardized β score	*P* value
Protein Connectivity	−0.068	0.003
3′ UTR length	−0.091	<0.001
Protein intrinsic disorder	0.101	<0.001
Singlish/multi interface hubs	−0.092	<0.001
RAP	−0.048	0.036
Gene Expression level	−0.035	0.097
Regulatory miRNAs number	−0.012	0.587

## Discussion

Profiling human neurodegenerative diseases from the perspective of protein evolutionary rates and comparing them with non-disease genes can provide therapeutic clues against disease pathogenesis. With this aim, we analyzed the evolutionary forces affecting NDD genes taking non-disease genes as the control one. We, thereby, found that higher selective pressure prevailed on NDD genes compare to the non-disease group. To explicate the reason behind such observation, we studied gene expression level, protein connectivity, regulatory miRNAs, disorder content, nature of hub proteins and relative aggregation propensity as evolutionary functions. In support of the conserved nature of NDD genes, we obtained higher gene expression level, higher protein connectivity along with greater miRNA regulation associated with them compare to the non-disease class. Interestingly, we observed lower disordered content of NDD genes contrasting previous publications [17], [18]. Moreover, the lesser disordered content of NDD genes underpin higher aggregation propensity of NDD genes due to lack of their conformational entropy [56], as reflected in our results. Emphasizing on the evolutionary rates differences between NDD and non-disease genes, our categorical regression model ascertained the independent influence of protein connectivity, presence of singlish/multi interface hub, protein disorderness, RAP and 3′UTR length among all the evolutionary parameters studied in this present analysis (Table 2).

**Figure 2:**
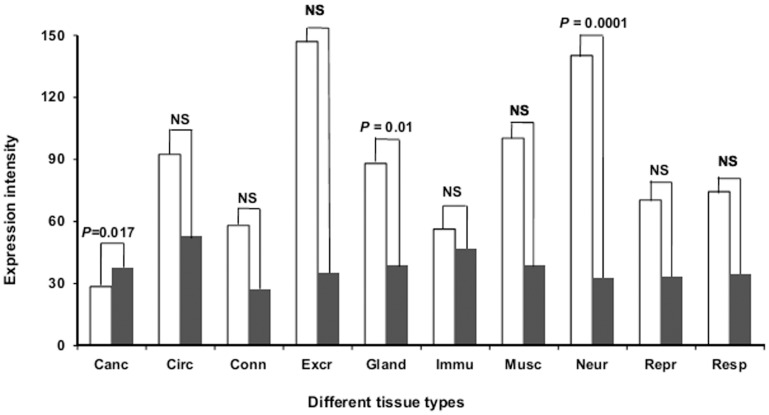
Expression profiles of NDD and non-disease genes considering 84 tissues in 10 major tissue categories. In this bar diagram, Cancerous, Circulatory, Connective, Excretory, Gland, Immune, Muscle, Neural, Reproductive and Respiratory tissues are abbreviated as Canc, Circ, Conn, Excr, Gland, Immu, Musc, Neur, Repr, and Resp respectively. The dark and light bars in each group represent non-disease and NDD genes respectively. From the picture, it is evident that our NDD genes are highly expressed in nervous system related tissues.

Our results share a conflicting view with Roychoudhury et al. [17] and Uversky [18] regarding disorder content of NDD proteins compare to the non-disease one. According to Roychoudhury et al. [17], NDD proteins are highly disordered proteins being “hub” in nature whereas we uttered about low disorderness of NDD proteins besides being hub proteins (Table 1). The disagreement in our result with Roychoudhury et al. [17] may arise due to the following reasons. In their analysis, Roychoudhury et al. [17] chose only three well-known neurodegenerative diseases (“Huntington”, “Parkinson” and “Alzheimer” diseases) as the representative of NDD group among all the different neurodegenerative diseases known at that time. However, for proper characterization of NDD proteins, it is essential to include all possible neurodegenerative diseases in the analysis. Since, our analysis highlights evolutionary rate difference between NDD and non-disease proteins, we only considered NDD proteins which have available evolutionary rate data. By this means, we collected 375 NDD proteins excluding neuropathies or lysosomal storage diseases and risk associated disease susceptible genes following extensive literature survey. Among them, 21.33% proteins (80 proteins out of 375 proteins) in our dataset overlapped with 352 NDD proteins selected by Roychoudhury et al. [17].

Proceeding further, we considered only overlapping 80 proteins as NDD group. In doing so, our comparative study established that NDD proteins share non significant (*P = *0.722) difference in disorder content with respect to non-disease group. From these results, we can conclude that the difference in gene selections may be a reason for obtaining such dissimilar result with Roychoudhury et al. [17]. Moreover, our in depth analysis revealed that NDD proteins are enriched with multi-interface hub (party hub) while the non-disease class are well populated with higher proportion of singlish-interface hub (date hub) (Figure 1). Since, multi-interface hubs promote simultaneous binding through their interaction domains compare to singlish-interface hubs, higher population of multi-interface hub in NDD category go in favor of their conserved nature. Hence, we proposed that the nature of “hub” was more important regulator of protein disorderness than hub content and thereby, protein evolutionary rates. However, Uversky [18] considered several case studies to demonstrate that intrinsically disordered proteins can easily form ordered hydrophobic β-sheet topology in contrast to folded globular proteins, required for fibril formation in aggregating proteins. Thus, he concluded that human aggregation prone neurodegenerative diseases are highly disordered proteins by nature. Regarding the aforementioned controversy with Uversky [18], we can say that by definition intrinsically disordered proteins lack any stable ordered secondary/tertiary structure under physiological conditions and prefers hydrophilic residues [13], [18]. In addition, intrinsically disordered positions in protein structures can not adopt any ordered structure and it is reasonable to assume that the crystal structures of those proteins do not contain any coordinate data of the atoms in these intrinsically disordered positions. On the other hand, β-sheet structures, a class of ordered secondary structures, have their coordinate data maintained in the X-ray crystal structures. Therefore, formation of β-sheet topology from intrinsically disordered proteins can contradict with their structural definitions. On a final note, we can say that being a positive evolutionary rate regulator [13], lower disorderness of NDD proteins in our dataset can completely describe the conserved nature of NDD proteins contrast to non-disease group.

From the perspective of gene expression level, our result supports Bortoluzzi et al. [60] for having higher gene expression level of human disease genes. On our way, we noticed that NDD genes are ∼2.54 fold highly expressed than non-disease class. Moreover, tissue expression breadth data also supports our result (Expression width of NDD genes = 5.37, non-disease genes = 2.16 and *P = *5.63×10^−18^ for NDD vs. non-disease genes). To obtain, a suitable reason for the elevated expression level for NDD genes, we checked the tissue distribution pattern of NDD genes compare to the rest of the non-disease group (Figure 2). Following Greco et al. [71], we classified 78 normal tissues into 9 major tissue categories and considered rest of the 6 abnormal tissues as “cancerous” group. In doing so, we obtained that except cancerous tissues, NDD genes share elevated expression level in all tissue types (Connective, Excretory, Gland, Immune, Muscle, Neural, Reproductive and Respiratory as shown in Figure 2). Since, our primary focus is on neurodegenerative diseases, our analysis (Figure 2) strongly supports the highest (*P* = 0.0001) expression of NDD genes near nervous system related tissues. In addition, we observed that our non-disease genes on average show uniform gene expression level within the range of 25–60 whereas, for NDD class the inhomogeneous expression level often fluctuates within the range of 25–150.

Molecular evolution is strongly fostered by genes’ efforts to avoid/tolerate errors while producing proteins. Besides identifying the evolutionary features of human neurological disorders, our investigation has clarified the complicated relationships between protein disorder content and RAP. Without these crucial informations, the ability to diagnose, prevent, and treat neurological disorders will remain incomplete.

## Supporting Information

Table S1 **List of human neurodegenerative disease genes and non-disease genes used in this study.**
(XLS)
